# The flexibility and convenience of using a standard orthopaedic table in peri-articular knee fractures: Allowing ligamentotaxis and improving imaging accessibility

**DOI:** 10.1016/j.cjtee.2022.01.005

**Published:** 2022-01-20

**Authors:** Zaid AL-Ani, Shaival S. Dalal, Amit Chandratreya, Khalid Sharif, Sarvpreet Singh

**Affiliations:** a Tauma & Orthopaedics, North West Anglia Trust, Hinchingbrooke Hospital, Huntingdon, United Kingdom; b Trauma and Orthopaedics, Princess of Wales Hospital, Bridgend, United Kingdom; c Trauma & Orthopaedic Diana Princess of Wales Hospital, Grimsby, North Lincolnshire, United Kingdom

**Keywords:** Fracture table, Ligamentotaxis, Tibial plateau, Distal femur

## Abstract

Tibial plateau and distal femoral fractures are common injuries presenting a significant operative challenge. Complexity of the fracture often needs multi-planar surgical access. A combined two-staged procedure is frequently suggested both in supine and prone position to address this issue. However, this will significantly increase the operative time and eventually impact the outcome, in addition to the complications associated with prone positioning. In this study we used a standard orthopaedic table to position these patients in order to grant access to the postro-medial and a postro-lateral structures while the patient stays in supine setup, at the same time, giving the flexibility to change the alignment from valgus to varus and vice versa. This facilitates fracture reduction while addressing the anatomical structure of the knee. A further advantage is the unobstructed imaging access throughout the surgical fixation. This facilitates the reduction in operative time hence leading to a better outcome in these difficult fractures. We tested this positioning technique in more than 40 patients over a 4-year period at two different centres in the United Kingdome. We found that this approach is safe, reproducible and relatively easy to set up in the two centres.

Tibia plateau and distal femoral fractures are common and debilitating lower limb injuries.^1^^,^^2^ For better joint function, reduced morbidity and better outcome, anatomic reduction to restore the articular surface and the longitudinal axis of the lower limb are essential.^3^^,^^4^ Complex and high velocity injuries are often challenging, even to highly skilled surgeons. Not uncommonly, the postro-lateral and postro-medial structures are also involved in these complicated tibia plateaus fractures.^5^ This is difficult to fix from the antro-lateral and antro-medial approach.^6^ Therefore surgeons often resort to changing the position of these patients to access the different fracture fragments.^7^ This procedure potentially increases the operative time and subjects the patient to a higher complication rate. A good patient positioning should give 360-degree access to the knee joint with good intraoperative imaging^5^, at the same time provides the convenience of maintaining reduction while fixing the fracture. This is even more important in the elderly patients where osteoporosis can complicate the fixation.

The surgical technique described here for the operative management of these fractures is based on using the principle of ligamentotaxis to aid reduction. Meanwhile a standard fracture table is used to position the patient in a way that allows an almost uninhibited access to all parts of the knee, thus requiring minimal use of an assistant, as well as facilitating convenient intraoperative imaging.

From January 2017 to January 2021, two different centres in the United Kingdome tested a modified patient positioning technique using standard locally available orthopaedic tables after sharing pictures and diagram of the assembly. This position was tested by different orthopaedic surgeons of different grades on 40 complex tibial plateau and distal femoral fractures. This positioning technique was tested for safety, efficiency, user-friendliness and whether it can reduce the overall time for fixation to improve outcome.

## Technique

A T30 Maquet orthopaedic operating table (usually used for hip fracture fixation) is prepared and assembled before anesthetising the patient to ensure availability of all parts needed for the procedure. A radiographer with C-arm should ideally be made familiar with the technique before starting the operation.

**Fig. 1:**
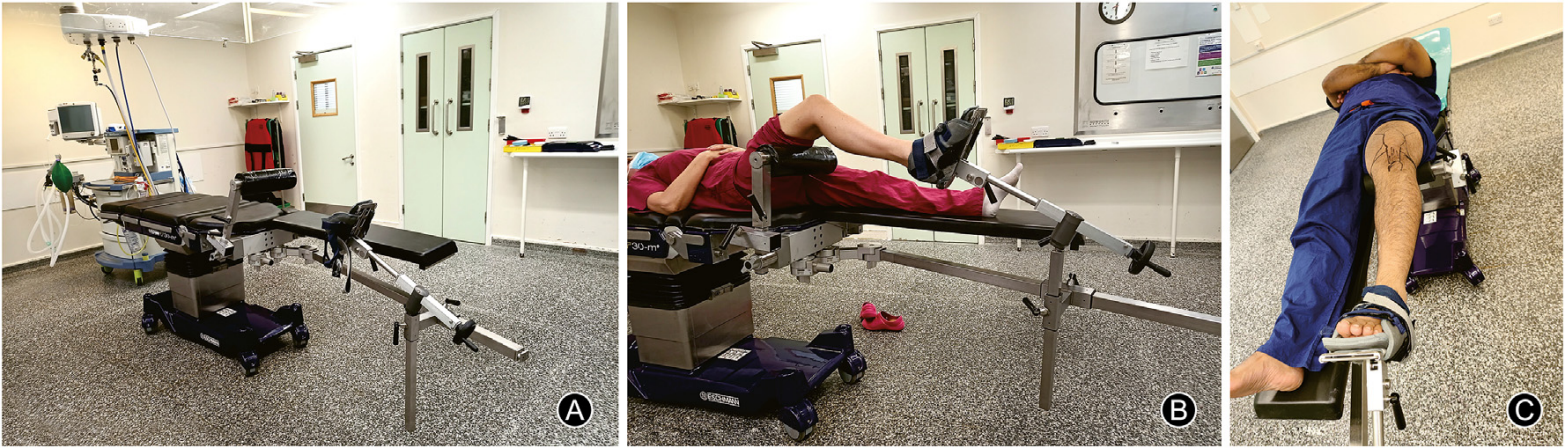
Operating table set up with attachments (A, B) and patient positioning with anatomical landmarks for the knee (C).

The attachments needed to achieve the intended position are as follows: (1) a transverse radiolucent post covered with a soft padded cylinder mounted on the side of the table (Fig. 1) (This is placed under the distal femur to lift the femoral condyles off the tibia in case of tibial plateau fractures, or on the back of proximal tibia in case of lower femur fractures. No hip post is used and the patient is put in slight Trendelenburg position for counter-traction), (2) a leg support for the non-operative leg, (3) a hinge joint with a metal bar, and (4) a foot support which is connected to the bar. These are standard attachments provided with the table.

The hinge joint and the connecting bar is mounted underneath the non-operative side and angulated towards the affected side; this setting initially feels counter-intuitive but is necessary to provide adequate space below the operation site for the C-arm rotation to allow the X-ray imaging during surgery.

**Fig. 2:**
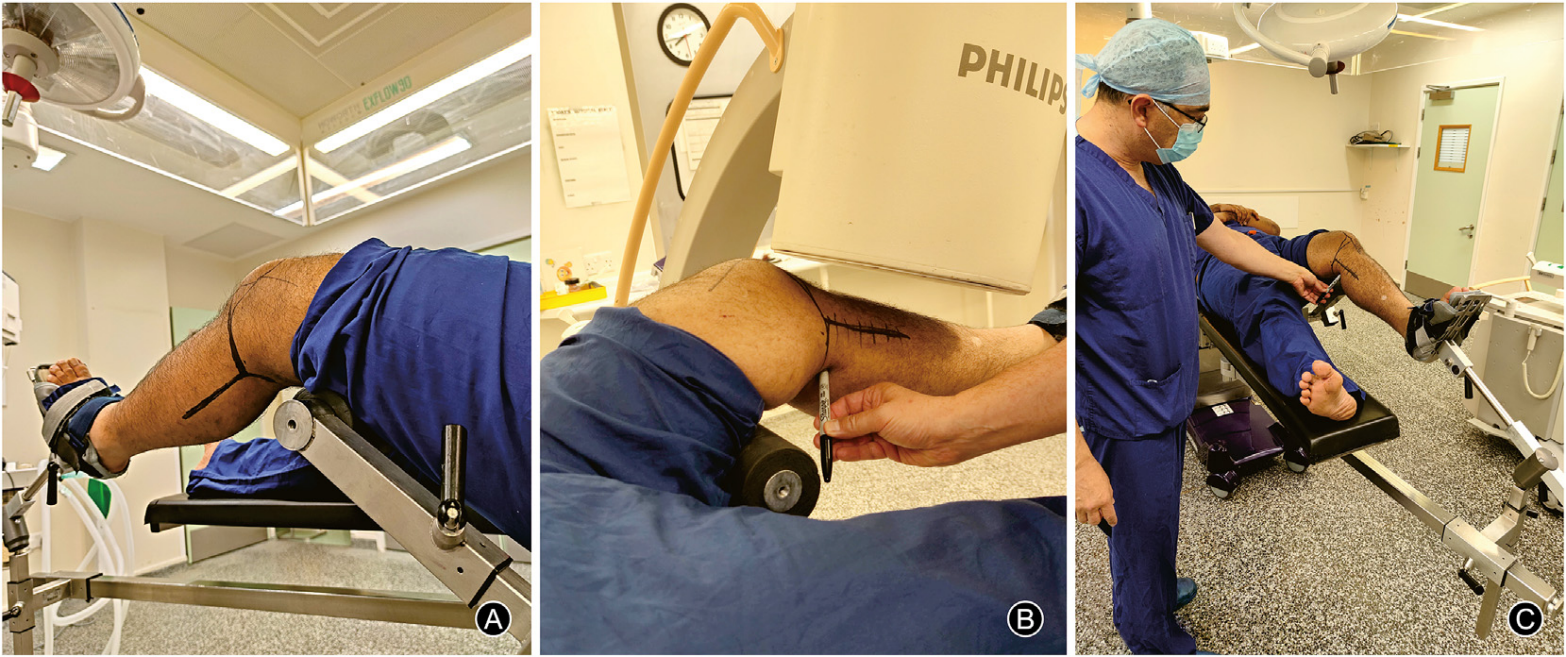
Patient positioning for upper tibia fractures with easy access to the postro-lateral part of the knee (A); and patient positioning showing X-ray approach for medial column fractures (B) as well as easy access to postro-medial structures patient in supine position (C).

After induction of anaesthesia, the patient is positioned supine on the operating table. Care should be taken that the bolster for lifting the femur is not positioned in the popliteal fossa but underneath the distal third femur to prevent pressure on the popliteal vessels (Fig. 2A). Limb alignment is restored through gentle traction and confirmed by an X-ray, before prepping and applying the surgical drapes. We can utilise a large Dynamic hip screw rape, which encompasses the leg completely, with the iodine impregnated part over the fracture site, which also gives good visual access to the entire limb.

In case of lateral column fracture, the C-arm will come across from the non-operative side with an angulation to insure a true anteroposterior (AP) view of the upper tibia. The surgeon will be standing next to the operating side. Operating on the medial column including postro-medial injuries, the surgeon will be standing next to the non-operative side (Figs. 2B and C). The C-arm in this situation will be coming from the operative side (Fig. 2B). Keeping the table at a higher level will ensure an easier access to the postro-medial structures. Lateral view is performed by rotating the C-arm under the bars holding the leg, vertical approach of the C-arm is recommended to create more room in this situation.

In case of distal femur fractures, the C-arm will approach from the non-operative side and angulated differently, to ensure a proper AP view of the distal femur. Lateral views are performed the same way in upper tibial fractures.

Moving the leg from valgus to varus helps restore the alignment, at the same time offloading the tibial plateau from the femoral condoyle. This will allow elevation of the lateral plateau.

## Discussion

Many techniques have been described to aid in reduction prior to fixation for tibia fractures, including manual traction, temporary distractors, temporary uni-cortical plating as well as table traction. None have proved superior.^9^ The traction techniques described for tibial plateau fractures rarely have the required versatility and easy imaging access, consequently there is no consensus regarding the best technique of fixing these fractures.^10^

The proposed positioning technique offers the flexibility and allows manipulation of the limb in both AP as well as medio-lateral plane due to the hinge joint attachment underneath the table. In cases of dual incisions, this can be started at the same time, further reducing surgical time. If needed, an arthroscopy can also be performed in this position, with some dexterity, to treat any chondral or meniscal pathology.

This technique keeps the knee at a relatively higher level to the rest of the body, thus providing a bloodless field and therefore the option of not using a tourniquet and hence reduces the disruption to the perfusion of already damaged and contused soft tissues often associated with these injuries. This is particularly important in complex cases requiring longer surgical time. Surgeons can still apply the tourniquet and inflate it only to control any accidental bleeding.

In depressed tibia plateau, having the flexibility of directing the traction from varus and valgus will ensure the right alignment; this will also lift the femoral condyles clear off the tibia aiding the reduction of the lateral tibial plateau to its convex contour. The sub-chondral bone void created by elevation of the lateral tibial plateau can then be filled with morselised bone chips or calcium phosphate granules for structural strength and for maintaining the lateral joint line elevation before definitive fixation. This flexibility is not possible in the available knee distraction procedures because of the fixed attachment.

We found this positioning technique to be very helpful in distal femoral fractures too. It allows traction along the femoral axis, while the knee is kept in a flexed position. This will counteract the deforming forces of gastrocnemius muscles and facilitates restoration to the distal femur alignment. Continuous mild traction throughout the procedure will provide relative stability while applying the implant. This is usually done through minimal access techniques. The whole procedure can be controlled clearly by an image intensifier, facilitating standardised AP and lateral intraoperative images comparable to the postoperative images, which is an added advantage of this technique over manual traction methods. Ligamentotaxis is a well-known technique for fracture reduction. Hammer et al. have described its use for distal femoral fracture fixation only but needed a special table.^11^

**Fig. 3:**
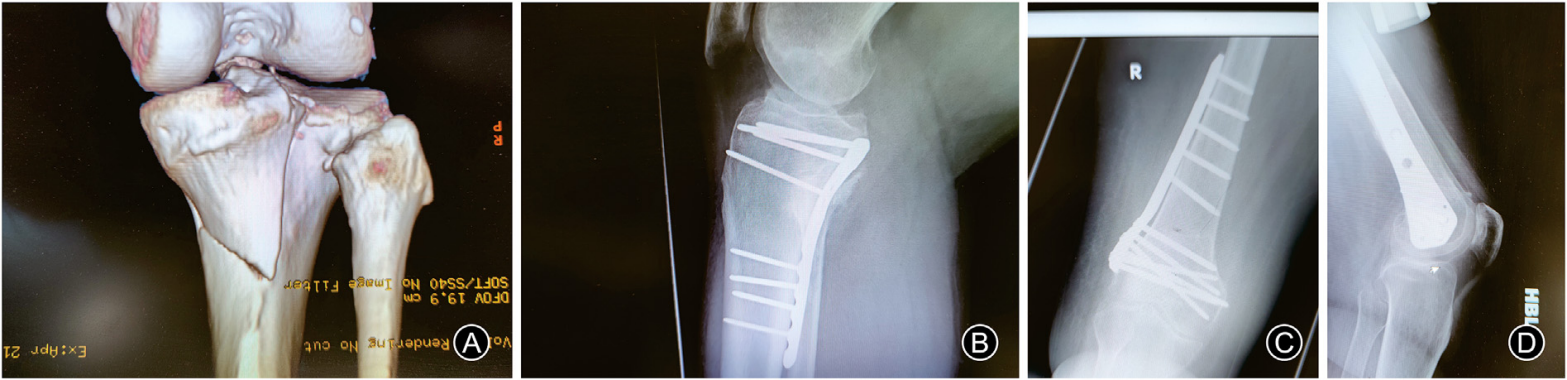
Preoperative (A) and postoperative (B) images of a posteromedial tibial fracture operated with posteromedial plating; preoperative (C) and postoperative (D) X-ray for another case of distal femur fractures.

**Fig. 4:**
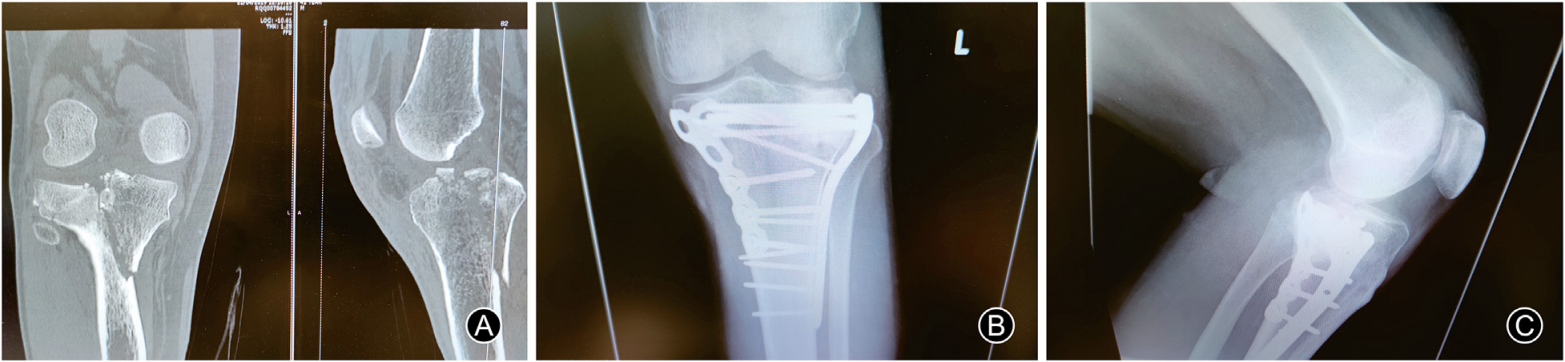
Preoperative (A) and postoperative (B, C) images of a bicondylar tibial fracture operated with dual plating. It shows the amount of distraction that can be achieved for successful reduction of the lateral depressed tibial articular surface.

The above-described technique has been used in two hospitals and for more than 40 cases (Fig. 3, Fig. 4) and was found reproducible and efficient in reducing the operating time and improving outcomes by all the surgeons in the two centres.

In conclusion, we found this technique useful in fracture reduction, improving access to the fracture site and providing ease of intra-operative imaging. An added advantage is that the operation can be performed without a tourniquet. Moreover, in cases requiring dual plating, the procedures can be started simultaneously on both sides. As the limb is fixed on a fracture table, it does not need frequent re-positioning for imaging in different planes. Because of these factors, we have found that using this technique has reduced our operating time for these complex and challenging injuries.

## Funding

Nil.

## Ethical statement

This positioning technique does not violate ethics while handling human tissue, all sections in the assembly are already authorised, tested and found safe to be used in Orthopaedic surgery.

## Declaration of competing interest

No conflict of interest was encountered in this study.

## Author contributions

The authors in this study are orthopaedic surgeons that work in two different centres in the UK who are involved in testing the positioning technique. The third author involved in writing and supervising the project. The first author designed the positioning technique and did write the article.
